# Plant Growth Regulators INCYDE and TD-K Underperform in Cereal Field Trials

**DOI:** 10.3390/plants10112309

**Published:** 2021-10-27

**Authors:** Matthew J. van Voorthuizen, Jiancheng Song, Ondřej Novák, Paula E. Jameson

**Affiliations:** 1 School of Biological Sciences, University of Canterbury, Christchurch 8140, New Zealand; matthewmjvv@gmail.com (M.J.v.V.); jcsong88@163.com (J.S.); 2 School of Life Sciences, Yantai University, Yantai 264005, China; 3 Laboratory of Growth Regulators, Institute of Experimental Botany of the Czech Academy of Sciences & Faculty of Science of Palacký University, CZ-783 71 Olomouc, Czech Republic; novako@ueb.cas.cz

**Keywords:** cytokinin, TD-K, thidiazuron, INCYDE, CPPU, isopentenyl transferase, IPT, cytokinin oxidase/dehydrogenase, CKX, wheat, barley, yield

## Abstract

Using plant growth regulators to alter cytokinin homeostasis with the aim of enhancing endogenous cytokinin levels has been proposed as a strategy to increase yields in wheat and barley. The plant growth regulators INCYDE and CPPU inhibit the cytokinin degrading enzyme cytokinin oxidase/dehydrogenase (CKX), while TD-K inhibits the process of senescence. We report that the application of these plant growth regulators in wheat and barley field trials failed to enhance yields, or change the components of yields. Analyses of the endogenous cytokinin content showed a high concentration of *trans*-zeatin (*t*Z) in both wheat and barley grains at four days after anthesis, and statistically significant, but probably biologically insignificant, increases in *cis*Z-*O*-glucoside, along with small decreases in *c*Z riboside (*c*ZR), dihydro Z (DHZ), and DHZR and DHZOG cytokinins, following INCYDE application to barley at anthesis. We discuss possible reasons for the lack of efficacy of the three plant growth regulators under field conditions and comment on future approaches to manipulating yield in the light of the strong homeostatic mechanisms controlling endogenous cytokinin levels.

## 1. Introduction

Food producers face a range of challenges in addressing global food security in the 21st century. These include continuing growth in food consumption in developing nations [1] and the effects of climate change, which will likely have significant and adverse effects on the environment and agriculture [2,3,4]. Increasing the yield of cereal crops, including wheat and barley, is fundamental to ensuring food security. In the 2019/2020 season, global production of wheat was more than 770 million tonnes, while for barley it was more than 150 million tonnes [5]. Several traits in cereals have been identified as important components of, and contributors to, overall yields, including having more productive tillers [6,7,8], a greater proportion of fertile grain-containing florets, larger grains, and leaf senescence occurring at an optimal time [9]. Notably, there can also be trade-offs between different components of yield, where increasing grain number can result in a decrease in grain weight [10,11,12,13,14]. Likewise, the production of more tillers is not necessarily beneficial, as small, unproductive tillers could direct resources away from productive tillers and negatively impact yield [15,16].

The cytokinins are a plant hormone group involved in many aspects of growth and development, including root and shoot growth [17,18,19], flower development [20,21], nitrogen signaling [22,23,24], senescence [25,26], stress response [27], seed yield components [28], and seed development [29,30,31,32], making them an important contributor to cereal yield.

Cytokinins are often grouped into three biologically active forms: naturally occurring, substituted adenines with either an *N*^6^ isoprenoid side chain or an aromatic side chain; and the synthetic diphenyl ureas. Briefly, isopentenyl transferase (IPT) catalyses the first committed step towards the formation of the isoprenoid cytokinins. The first formed cytokinins are the nucleotides that are converted by LOG (LONELY GUY) to the active free base forms, *trans*-zeatin (*t*Z), *N*^6^-isopentenyladenine (iP), *cis*-zeatin (*c*Z), and dihydrozeatin (DHZ), which are detected by a two-component signaling system. Cytokinin levels are controlled through destruction by cytokinin oxidase/dehydrogenase (CKX) or inactivation by cytokinin glucosyl transferase to *O*- or *N*-glucosides [32].

Previous attempts at manipulating yield and endogenous cytokinin have included the direct application of cytokinin itself to both wheat [33,34] and barley [35,36]. These approaches have involved direct injection into plant organs [34,37,38] or, more practically, through irrigation and spraying ([39], and references therein). However, success in field trials has been mixed, with findings in controlled experiments often hard to replicate in the field given the range of environmental factors and the complexity of analyzing their effects [12,39,40].

An alternative to the application of cytokinin has been the targeting of the enzymes that either deactivate cytokinin through glucosylation [41], or irreversibly degrade cytokinin via CKX [42,43]. Targeting *CKX* expression and/or activity has been suggested as a potential strategy to enhance yield [28,31,44,45,46], and *CKX* gene family members (GFMs) have been identified as being important for determining yield in both wheat and barley ([9], and references therein).

Given the challenge of increasing yield in the field using cytokinin [31], there has been a search for alternative compounds that might impact components of yield, including compounds that target CKX and compounds that might affect yield through other processes, including senescence. Such compounds include CPPU, TDZ, and the novel plant growth regulators (PGRs) INCYDE and TD-K [46,47,48,49]. These compounds became the focus for this research.

**Figure 1:**
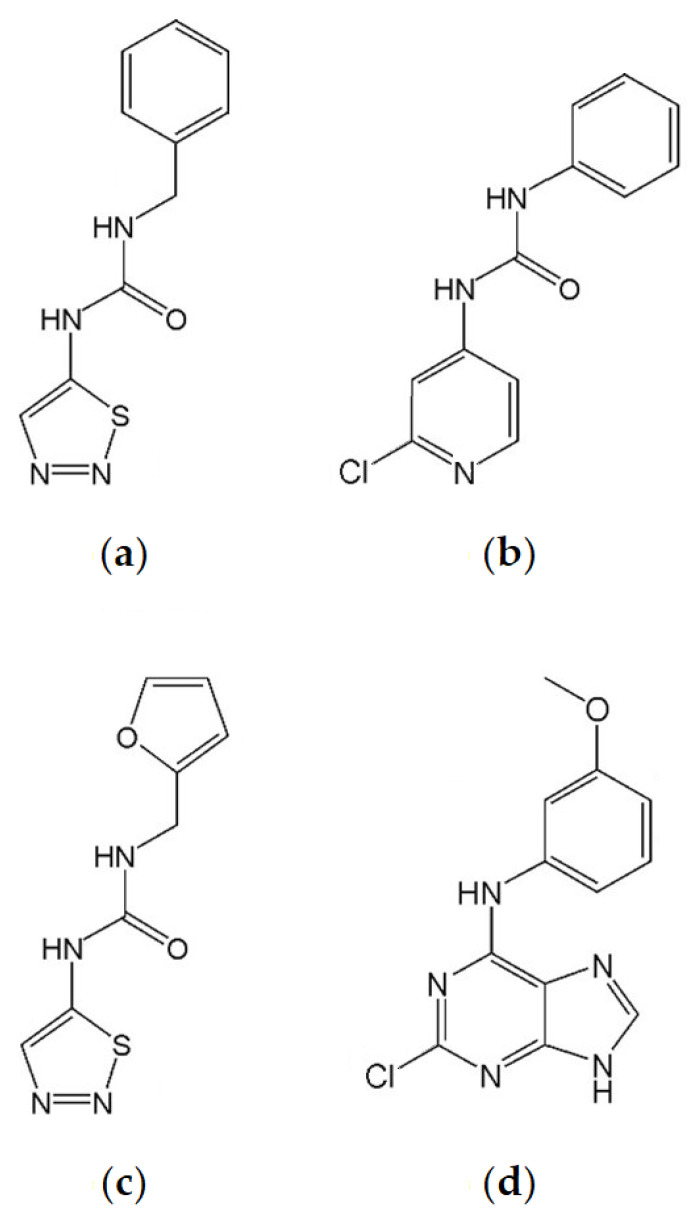
Structures of plant growth regulators. (**a**) Thidiazuron. (**b**) CPPU. (**c**) TD-K. (**d**) INCYDE.

Thidiazuron is a substituted phenylurea (Figure 1a) that has been shown to inhibit CKX [50,51,52]. Thidiazuron has strong cytokinin activity [53,54,55]. It is able to activate cytokinin receptors [48,53,54,56] and has anti-senescence properties [46,57] that are stronger than *trans-*zeatin *(t*Z) and 6-benzylaminopurine (BAP) [48]. It is also able to promote shoot growth [58,59,60,61], increase fruit size [62], and produce ethylene when applied to leaves [48]. The latter property makes it desirable as a cotton defoliant [63].

CPPU (*N*-(2-chloro-4-pyridyl)-*N*’-phenylurea) is a diphenylurea derivative (Figure 1b) which is able to inhibit CKX [64,65] more strongly than TDZ [66]. Although it activates cytokinin receptors AHK3/AHK4, it does so more weakly than TDZ [52]. CPPU is also reported to be able to delay senescence [67], promote shoot formation [68], enhance fruit size [69,70,71,72], promote earlier flowering [73], and provide resistance to drought stress [74].

TD-K (*N*-furfuryl-*N*’-1,2,3-thiadiazol-5-yl-urea) is a diphenylurea thidiazuron derivative (Figure 1c) which has strong cytokinin activity comparable to BA in *Amaranthus* and tobacco callus bioassays [49]. TD-K has strong anti-senescence capacity, relative to TDZ and BA [48,49]. Compared to TDZ, it more weakly activates cytokinin receptors [49,53,54,56], is less able to promote ethylene production in mung bean hypocotyls [75], and, in contrast to TDZ, does not inhibit root growth [48].

INCYDE (2-chloro-6-(3-methoxyphenyl)aminopurine) is a substituted 6-anilinopurine derivative (Figure 1d). It is a stronger inhibitor of cytokinin oxidase/dehydrogenase than TDZ, while more weakly activating cytokinin receptors compared to TDZ and *t*Z [55]. It activated the cytokinin responsive reporter gene *ARR5:GUS* [76] in a dose-dependent manner. INCYDE was shown to enhance yield of Rapid Cycling *Brassica rapa* but only under specific, controlled conditions [49]. INCYDE increased shoot FW in *CKX1*-overexpressing *Arabidopsis thaliana* seedlings [76]. INCYDE application has been reported to increase flower production in tomatoes [77], shoot production when applied with BA [78], and has a dose-dependent inhibition of shoot and/or root growth in *Bulbine natalensis* and *Rumex crispus* [45] and micropropagated *Eucomis autumnalis* [78]. INCYDE is also reported to alleviate the effects of biotic [79] and abiotic stress [45,77]. Additionally, when applied in the field to barley, analogue INCYDE-F was responsible for altering the endogenous cytokinin content [80].

Three PGRs with different properties and modes of action were selected for this investigation: INCYDE, TD-K, and CPPU. These compounds were applied to wheat and barley in field trials and components of the yields were analyzed. The effects of these compounds on endogenous cytokinins was also examined.

## 2. Results

### 2.1. Field Trial Analyses

Analyses carried out on the harvested wheat and barley from the field trials did not reveal any statistically significant difference in the yield (T/ha), thousand grain weight (TGW) in grams (g), or protein composition between any of the treatments and the controls for either wheat or barley (Table 1). The Orator wheat (2013/14) field trial was broadly infected with *Septoria* during a critical time in development, which negatively impacted the yield. Given the lack of evidence for any change in yield, the field trials were discontinued. Additional trials were carried out using outdoor pot trials where the same treatments and growth stages described for the field trials were used, but no statistically significant differences in yield or yield components were found for these trials either [81].

### 2.2. LC–MS/MS Analyses in Grain

LC–MS/MS analyses of wheat and barley grains from control plants assessed four days after anthesis (4 DAA) show that the concentration of *t*Z was much greater than the concentration of the other free bases iP, *c*Z, or DHZ (Table 2 and Table 3). Inactivation by glucosylation is clearly evident, as shown by the elevated levels of *c*Z- and *c*Z riboside-*O*-glucosides (*c*ZOG and *c*ZROG) in barley, and in wheat by elevated levels of *t*Z 9-glucoside (*t*Z9G), *c*ZOG, and *c*ZROG.

In wheat grains, neither TD-K nor CPPU treatment resulted in a significant change in any of the cytokinin metabolites compared to the control (Table 2). At four days following INCYDE treatment/anthesis in barley grains, there was a significant increase in the content of *c*Z *O*-glucoside (*c*ZOG), *c*Z-types overall and the total *O*-glucoside cytokinins (Table 3). Conversely, there were small but statistically significant decreases in the concentration of *c*ZR, DHZ, DHZR, DHZOG, and the total base and ribosides of *c*Z and DHZ cytokinins following INCYDE application.

## 3. Discussion

The region where our field trials were conducted, Canterbury, New Zealand, is known for world record cereal production (17.398 tonnes per hectare of wheat crop (Guinness World Records, 2020)). Our trials were conducted under optimal field conditions of water and fertilizer, which we recognize as a potentially challenging environment to assess PGR efficacy, a comment also made by Nisler et al. [66] with respect to their PGR field trials in the Czech Republic.

The lack of yield enhancement following INCYDE application (Table 1) suggests that this compound had little effect in our field trials on either wheat or barley. Positive trends in yield in field trials of wheat and barley treated with cytokinin derivatives similar to INCYDE have been reported but these failed to reach statistical significance [80]. Consequently, our field data are not in conflict with this. While the Orator wheat (2013/14) field trial, where INCYDE was applied, was impacted by *Septoria,* there was no evidence of INCYDE ameliorating the effect of this disease, in contrast to the report by Reusche et al. [79]. This is not to imply that INCYDE is not efficacious under other conditions, as changes in gene expression occur following application [83], and responses are clearly evident under more controlled environments, including in bioassays [55], in in vitro culture settings [45,77,78,79], and in pot trials with Rapid Cycling *Brassica rapa* [49].

The statistically significant increase in *c*ZOG following INCYDE application to barley may show a mechanism in common with previous in vitro experiments, where INCYDE (with BA) enhanced *O*-glucoside accumulation in banana plantlets [84]. It is possible that active cytokinin forms may have been channelled into inactivated *O*-glucosides as a consequence of reduced inactivation by CKX, due to inhibition of CKX by INCYDE. Because of the activation of homeostatic mechanisms, and also because of the very high endogenous levels of active *t*Z immediately after anthesis, any transitory increases in active cytokinins, if they had occurred, are likely to be biologically insignificant.

Neither of the two diphenylurea-derivatives, TD-K or CPPU, enhanced yield (Table 1). This is in contrast with an increase of 120.9% for oilseed rape yield (6.038 vs. 4.99 T/ha), and 106% (7.02 vs. 7.49 T/ha) for spring barley reported in the TD-K patent for PGR application at BBCH50 (extension growth) [48]. Details of statistical significance are not, however, provided for the different crops. More recently, a diphenylurea derivative was applied to barley and wheat under field conditions in the Czech Republic [66]. Although these studies targeted earlier growth stages, including at BBCH 20–25, as well as seed treatments, they also targeted the emergence of the inflorescence (BBCH 51), and at a concentration range between 5 and 50 µM, which is comparable to that used in our study. However, the field data for wheat and barley treated with diphenylurea-derivative Compound 19 are only presented as percent of control without statistical analyses available [66]. The variability apparent between years (particularly in tiller number and 1000 grain weight) makes it essential that statistical analysis of the yield data (0.7 to 6.6% yield increase compared to control) is presented.

Likewise, CPPU, despite having success with enhancing fruit size, has not had much success when used to target cereals in the field ([32], and references therein). The difficulties of replicating findings from controlled environments onto the field have been reported [12,40], with field trials introducing a multitude of uncontrolled or difficult to control factors, many of which could affect cytokinin homeostasis.

An increased tiller number is not necessarily seen as desirable in wheat [9], so we specifically targeted the PGRs at later stages of development: for TD-K this was from anthesis onwards, due to its strong anti-senescence properties [48,49]; and for INCYDE and CPPU from GS39, when florets are being established, and/or GS51, when ears are particularly susceptible to stress [85,86], and or across anthesis, the latter chosen due to the rapidly changing cytokinin content and elevated *CKX* expression associated with this stage in development ([9], and references therein). Indeed, a high level of *t*Z cytokinin was identified in wheat four days after anthesis (DAA) (Table 2). This aligns with previous reports of high levels of zeatin in wheat early in grain development [87,88,89,90,91], and, moreover, confirms that this cytokinin is *t*Z. The transient nature of this narrow developmental window that is associated with cell division is also a possible reason for the lack of yield enhancement by cytokinins in cereal field trials ([31], and references therein), since in the field environment, anthesis is spread across several days, although we attempted to cover this by applications at GS61 and 65.

The high concentration of *t*Z in barley at 4 DAA (Table 3) has also been reported [92]. In contrast, the low concentration of *c*Z contrasts with the high peak of *c*Z reported previously in developing barley kernels [93]. This suggests that 4 DAA is possibly after the *c*Z peak. The high concentration of *c*ZOG suggests active deactivation of *c*Z within days post-anthesis.

Our research suggests that INCYDE, TD-K, and CPPU have little to no effect on components of harvestable yield in wheat and barley grown under optimal field conditions. Additionally, this research highlights some of the difficulties and issues of conducting field trials with PGRs, with any attempt to manipulate cytokinin made more difficult not only by strong homeostatic responses but also by the complex, pleiotropic nature of cytokinin [31,39]. Feedback responses following the disturbance of cytokinin homeostasis have been observed or suggested elsewhere in the form of an increase in *CKX* expression and/or activity [14,94,95,96,97,98,99]. An increase in cytokinin following CKX inhibition might also be responsible for an enhancement in the deactivation of cytokinins, which could explain the stronger production of *cis*-type *O*-glucosides seen in barley (Table 3). Feedback mechanisms might also involve *IPT* GFMs, with *HvIPT1* and *HvIPT2* both being downregulated in response to a local increase in cytokinin following the knockout of *HvCKX1* [100].

Despite these difficulties, targeting CKX is still an important strategy for manipulating cytokinin and yield [9,31,45,46], and arguably more suitable than alternative strategies, including the direct application of cytokinin, or targeting IPT, given that CKX is considered a more moderate or ‘softer’ regulator of cytokinin compared to IPT [101]. Future research could focus on determining if the endogenous changes in barley (Table 3) and, indeed, the lack of change in wheat, were the result of changes in expression of genes associated with cytokinin homeostasis, including biosynthesis (*IPTs*), degradation (*CKX*s), and glucosylation (*CGTs*), and whether these results could help explain the lack of yield in the field trials. Additionally, with the identification of the key *CKX* gene family members that affect yield in wheat (reviewed in [9]), and with interesting results in wheat [13,14,102], barley [12,87,103,104], and rice [44,105] trials, transgenic approaches hold significant potential for enhancing yield in cereals.

However, whether the resulting cereal is a result of genetic modification or gene editing, in some jurisdictions such plants are subject to legal and social restrictions which make their cultivation, processing, and marketing difficult or impossible [106,107,108]. In this context, non-transgenic approaches, such as the Targeting Induced Local Lesions In Genome (TILLING) strategy, offer numerous advantages, including overcoming the limits imposed by the lack of genetic variability in traditional breeding, the acceleration of breeding programs, and, above all, the possibility of developing new varieties that do not have the limitations that characterize transgenic organisms [107]. Both the CRISPR/Cas9-mediated gene editing technology and the TILLING approach have their own merits and demerits relating to the initial investment by researchers, the access to the requisite technology, the range of mutations that are either targeted (in gene editing) or identified (multiple point mutations in TILLING) and their use in breeding [106].

More recently, two in silico TILLING resources have been generated and made publicly available. These include the whole exome sequencing of over 1200 TILLING mutant lines of a well-known European bread wheat variety Cadenza [109,110]. Similarly, an in silico TILLING resource is being generated for the most widely grown Chinese bread wheat variety, Jimai 22. Within this population, multiple point mutants for not only all *CKX* GFMs but also the zeatin *O*-glucosyl transferase (*ZOGT*) GFMs have been identified [9,41]. Importantly, while *CKX* GFMs have been the target of much research [9], the high levels of cytokinin glucosides in wheat and barley, and the negative relationship of *ZOGT* gene expression with yield in wheat [41,91], indicate that the *ZOGT* GFMs warrant further investigation, which is beyond the tools offered by the CKX inhibiting PGRs.

## 4. Materials and Methods

### 4.1. Field Trials

Wheat and barley field trials were carried out over two seasons, near Lincoln, New Zealand (43°36′15.7″ S 172°25′56.0″ E and 43°37′04.7″ S 172°27′09.4″ E). Autumn-sown wheat (cultivar Orator) was grown in the 2013/14 season, while barley (cultivar Quench) and wheat (cultivar Torch) were grown in 2014/15. Sowing spacing was kept constant, to prevent any confounding effect on tiller number. Field trials were carried out in a farmer’s paddock and subject to standard field management including regular irrigation, fertilizer application, and application of compounds, including herbicide, insecticide, and fungicides, where necessary. Field trials were planted in 10 m × 2.5 m plots, arranged in a randomized complete block design with four replicates for each treatment. Plant growth regulators INCYDE, TD-K, and CPPU were applied at concentrations between 10 and 100 µM at growth stages (GS), defined according to the Zadoks scale [82], including GS 39 (the appearance of flag leaf ligule), GS 51 (appearance of the spikelet), and at GS 61 to 69 (defined as anthesis). Plant growth regulators were applied at rates of 187 L/ha for the 2013/14 trial, and 170 L/ha for the 2014/15 trial.

INCYDE, TD-K and CPPU were prepared by dissolving compounds in dimethylsulfoxide (DMSO) (Scharlab), diluted with water and then, prior to application, mixed with surfactant (Yates Sprayfix, Yates) at 0.5% (*v/v*). Two controls were used in the field trials, ‘untreated controls’ where no application was made, and ‘DMSO controls’ where the amount of DMSO used was equivalent to the highest PGR concentration for each respective trial, unless stated otherwise in the results. Applications were made by New Zealand Arable using CO_2_ pressurized hand-hand plot booms for applications rates between 170–190 L/ha.

### 4.2. Plant Material

#### 4.2.1. Yield and Protein Composition Analyses

Once wheat and barley plants had senesced completely, plants were harvested with a Sampo combine harvester (Sampo Rosenlew Ltd., Pori, Finland) and protein content was analyzed by New Zealand Grainlab. Onboard weighing provided the analysis of yield (tonnes per hectare) and, using 20 g screened samples of grain, the TGW was calculated with a Numigral I seed counter (Sinar). Protein composition was analyzed using an Instalab^®^ 700 NIR Analyzer (DICKEY-john). The thousand grain weight was calculated for each plot, using 20 g of screened grain samples.

#### 4.2.2. LC–MS/MS Analyses

Grain material for LC–MS/MS analyses was sampled from the field trials, following anthesis-targeted application of either INCYDE (50 µM), TD-K (50 µM), CPPU (100 µM), or water + DMSO. Following treatment, whole heads were sampled at day 4 after anthesis, which was 4 days after treatment. Wheat and barley heads were frozen by immediately submerging the samples in liquid nitrogen and storing at −80 °C. Wheat and barley grains were dissected from the middle third section of the spike, with basal florets within the spikelet targeted in wheat [9]. Grains were then organized, based on the developmental stages as described in [111]. INCYDE-treated wheat grains were not sampled for LC–MS/MS analyses, given that this trial (wheat cv. Orator, 2013/14) was infected with *Septoria* at a critical time during grain development.

Grains were ground under liquid nitrogen and freeze dried with a Savant™ SPD131DDA SpeedVac™ Concentrator (Thermo Fisher Scientific) to produce samples weighing between 8 to 22 mg. For each treatment, three replicates were prepared. Samples were then analyzed according to [112]. Sample extraction was carried out with a modified Bieleski solution (60% MeOH, 10% HCOOH, and 30% H_2_O), and [^13^C_5_]*c*Z, [^13^C_5_]*t*Z, [^2^H_5_]*t*ZR, [^2^H_5_]*t*Z7G, [^2^H_5_]*t*Z9G, [^2^H_5_]*t*ZOG, [^2^H_5_]*t*ZROG, [^2^H_5_]*t*ZMP, [^2^H_3_]DHZ, [^2^H_3_]DHZR, [^2^H_3_]DHZ9G, [^2^H_7_]DHZOG, [^2^H_3_]DHZMP, [^2^H_6_]iP, [^2^H_6_]iPR, [^2^H_6_]iP7G, [^2^H_6_]iP9G, [^2^H_6_]iPMP stable isotope-labelled standards (0.25 pmol of cytokinin bases, ribosides, *N*-glucosides, 0.5 pmol of cytokinin *O*-glucosides and nucleotides; Olchemim) were added to each sample to validate phytohormone determination. Sample purification was carried out with mixed-mode cation-exchange (MCX) cartridges (Oasis MCX, 30 mg/1 mL; Waters). Analytes were eluted by two-step elution using a 0.35 M NH_4_OH aqueous solution and 0.35 M NH_4_OH in 60% (*v/v*) methanol solution. The resulting eluate was subsequently evaporated to dryness and then dissolved in the mobile phase (15 mM ammonium formate pH 4.0 in 5% (*v/v*) methanol). LC–MS/MS analyses were carried out using a Acquity UPLC^®^ System (Waters) and a triple quadrupole mass spectrometer Xevo^TM^ TQ MS (Waters). The mass spectrometry data was then processed utilizing MassLynx™ Mass Spectrometry Software with TargetLynx™ (Waters).

### 4.3. Statistical Analyses

For yield and protein composition from the field trials, the mean was generated using four replicates for each treatment and the data presented with standard errors. Statistically significant differences, where *p* ≤ 0.05, were determined between PGR treatments and the respective DMSO control using a two-way ANOVA. A logit transformation was made to protein composition data prior to ANOVA analysis. Similarly, statistically significant differences for LC–MS/MS data were determined between PGR treatments and the control using two-way ANOVA (significance level: 0.05), with a *post hoc* two-sided Dunnett test (Confidence Interval: 95%). To ensure the assumptions of the ANOVA were met, an examination of Q-Q plots of standardized residuals was made, and where necessary the equality of variances ensured through a Levene’s test and plot of standardized residuals and predicted values.

## Figures and Tables

**Table 1:** Yield and protein composition in wheat (cv. Orator and cv. Torch) and barley (cv. Quench).

**Wheat Cultivar Orator (2013/14)**			
**Treatment**	**Yield (T/ha)**	**TGW (g)**	**Protein (%)**
Untreated Control	10.8 ± 0.2	46.0 ± 1.1	11.2 ± 0.2
DMSO 50 µM Control (GS 39, 51, 61, 65)	11.1 ± 0.1	46.6 ± 0.2	11.2 ± 0.1
DMSO 25 µM Control (GS 61, 65, 65 + 13 d)	11.2 ± 0.1	47.2 ± 0.7	11.0 ± 0.1
INCYDE 10 µM (GS 65)	11.4 ± 0.2	45.9 ± 1.1	11.4 ± 0
INCYDE 25 µM (GS 39, 51, 61, 65)	11.3 ± 0.2	46.2 ± 0.7	11.1 ± 0.1
INCYDE 25 µM (GS 39)	11.2 ± 0.2	45.0 ± 1.1	11.2 ± 0.1
INCYDE 25 µM (GS 51)	11.0 ± 0.1	45.4 ± 0.8	11.1 ± 0.2
INCYDE 25 µM (GS 61)	11.1 ± 0.2	45.6 ± 0.4	11.1 ± 0.1
INCYDE 25 µM (GS 65)	11.1 ± 0.2	45.1 ± 0.5	11.2 ± 0.1
INCYDE 50 µM (GS 61)	11.1 ± 0.1	45.5 ± 1.2	11.4 ± 0.1
INCYDE 50 µM (GS 65)	11.1 ± 0.2	46.9 ± 0.3	11.4 ± 0.1
TD-K 10 µM (GS 61, 65, 65 + 13 d)	11.1 ± 0.1	46.3 ± 1.1	11.1 ± 0.1
TD-K 25 µM (GS 61, 65, 65 + 13 d)	11.4 ± 0.1	46.0 ± 0.5	11.1 ± 0.1
**Wheat Cultivar Torch (2014/15)**			
**Treatment**	**Yield (T/ha)**	**TGW (g)**	**Protein (%)**
Untreated Control	14.5 ± 0.1	46.8 ± 1.1	9.8 ± 0.2
DMSO Control (GS 51, 61, 65, 65 + 15 d)	14.5 ± 0.3	48.1 ± 0.2	9.9 ± 0.2
TD-K 10 µM (GS 61, 65, 65 + 15 d)	14.5 ± 0.2	47.4 ± 0.5	10.0 ± 0.2
TD-K 50 µM (GS 61, 65, 65 + 15 d)	14.7 ± 0.3	49.1 ± 0.5	9.8 ± 0.05
CPPU 10 µM (GS 61, 65)	14.4 ± 0.3	47.1 ± 0.4	10.0 ± 0.2
CPPU 30 µM (GS 61, 65)	14.5 ± 0.3	48.8 ± 0.2	9.7 ± 0.1
CPPU 100 µM (GS 61, 65)	14.6 ± 0.2	48.4 ± 0.3	9.7 ± 0.3
CPPU 10 µM (GS 51, 65)	14.5 ± 0.2	47.8 ± 0.4	9.6 ± 0.1
CPPU 30 µM (GS 51, 65)	14.7 ± 0.1	47.2 ± 0.9	9.9 ± 0.1
CPPU 100 µM (GS 51, 65)	14.4 ± 0.2	48.1 ± 0.8	9.9 ± 0.2
**Barley Cultivar Quench (2014/15)**			
**Treatment**	**Yield (T/ha)**	**TGW (g)**	**Protein (%)**
Untreated Control	10.8 ± 0.2	52.3 ± 0.5	14.3 ± 0.1
DMSO Control (GS 51, 61, 65, 65 + 15 d)	11.0 ± 0.1	52.0 ± 0.4	13.9 ± 0.4
INCYDE 10 µM (GS 65)	11.0 ± 0.2	53.0 ± 0.2	13.8 ± 0.3
INCYDE 25 µM (GS 39, 51, 61, 65)	11.2 ± 0.2	51.8 ± 0.5	14.0 ± 0.2
INCYDE 25 µM (GS 39)	11.3 ± 0.2	52.5 ± 0.5	13.8 ± 0.2
INCYDE 25 µM (GS 51)	11.3 ± 0.1	52.4 ± 0.9	14.0 ± 0.4
INCYDE 25 µM (GS 61)	11.1 ± 0.1	52.2 ± 0.9	13.9 ± 0.5
INCYDE 25 µM (GS 65)	11.3 ± 0.1	52.5 ± 0.7	14.2 ± 0.4
INCYDE 50 µM (GS 61)	11.2 ± 0.1	52.5 ± 0.8	14.1 ± 0.4
INCYDE 50 µM (GS 65)	11.2 ± 0.3	52.3 ± 0.4	13.9 ± 0.5

Data were analyzed using an ANOVA, with protein percentage data logit-transformed prior to ANOVA. Data are presented as the means ± standard error (*n* = 4). Yield is provided in tonnes per hectare (T/ha), thousand grain weight (TGW) in grams (g) and protein as a percentage (%). Concentration of each treatment is given in µM, with growth stage (GS) indicating the growth stage (Zadoks scale [82]) targeted for treatment, and ‘d’ indicating the number of days after the respective growth stage. The dimethylsulfoxide (DMSO) controls list the GS targeted, with volumes equivalent to the DMSO used in the highest concentration within each field trial, with the exception of Orator (2013/14), where DMSO Control (GS 61, 65, 65 + 13 d) was provided at a volume equivalent to 25 µM applications.

**Table 2:** LC–MS/MS analyses of the quantity of cytokinins in wheat (cultivar Torch, 2014/15) grains treated at anthesis with TD-K or CPPU. Measurements were made at four days after anthesis.

	Wheat Cytokinin Concentrations (pmol/g DW)		
Type	Control	TD-K 50 µM	CPPU 100 µM
*t*Z	794.5 ± 71.1	705.3 ± 75.7	889.2 ± 73.7
*t*ZR	60.0 ± 6.7	60.3 ± 3.2	63.5 ± 2.3
*t*ZOG	20.4 ± 2.8	24.7 ± 0.5	22.8 ± 1.3
*t*ZROG	5.8 ± 0.7	6.8 ± 0.2	6.5 ± 0.5
*t*ZRMP	115.9 ± 3.6	106.5 ± 2.8	103.1 ± 10.5
*t*Z7G	<LOD	<LOD	<LOD
*t*Z9G	247.3 ± 21.3	286.1 ± 3.5	268.7 ± 11.5
Total *t*Z types	1244.0 ± 104.8	1189.7 ± 85.2	1353.8 ± 57.0
iP	1.6 ± 0.2	1.3 ± 0.1	1.3 ± 0.1
iPR	2.2 ± 0.2	1.8 ± 0.1	2.5 ± 0.4
iPRMP	22.9 ± 1.5	22.1 ± 3.8	25.9 ± 4.0
iP7G	<LOD	<LOD	<LOD
iP9G	<LOD	<LOD	<LOD
Total iP types	26.8 ± 1.7	25.1 ± 3.9	29.7 ± 4.1
*c*Z	9.6 ± 0.5	7.8 ± 1.1	8.6 ± 1.2
*c*ZR	33.9 ± 3.9	24.7 ± 3.5	30.5 ± 4.0
*c*ZOG	114.3 ± 12.4	130.8 ± 11.7	116.1 ± 12.9
*c*ZROG	139.1 ± 13.2	154.5 ± 6.6	150.6 ± 8.9
*c*ZRMP	10.0 ± 0.8	7.7 ± 1.3	10.8 ± 1.2
*c*Z9G	<LOD	<LOD	<LOD
Total *c*Z types	306.5 ± 26.5	325.5 ± 12.2	316.5 ± 17.0
DHZ	0.23 ± 0.01	0.20 ± 0.03	0.20 ± 0.03
DHZR	2.9 ± 0.1	2.4 ± 0.3	2.6 ± 0.3
DHZOG	1.4 ± 0.2	1.7 ± 0.1	1.5 ± 0.1
DHZROG	9.5 ± 1.1	10.7 ± 0.6	10.0 ± 0.8
DHZRMP	<LOD	<LOD	<LOD
DHZ7G	15.0 ± 0.3	13.3 ± 0.9	14.6 ± 2.2
DHZ9G	0.07 ± 0.003	0.06 ± 0.01	0.07 ± 0.01
Total DHZ types	29.1 ± 1.5	28.2 ± 0.7	28.9 ± 2.6
Total CK bases	806.0 ± 71.4	714.6 ± 76.9	899.3 ± 74.9
Total CK ribosides	98.7 ± 6.8	89.1 ± 7.0	99.0 ± 7.0
Total CK nucleotides	148.9 ± 3.7	136.3 ± 2.0	139.8 ± 12.8
Total CK *O*-glucosides	290.5 ± 30.2	329.2 ± 18.8	307.5 ± 23.7
Total CK *N*-glucosides	262.3 ± 21.6	299.4 ± 3.9	283.4 ± 13.6
Total cytokinins	1606.4 ± 117.3	1568.5 ± 69.5	1728.9 ± 43.4

Treatments were compared to the control using a two-sided ANOVA. Data are presented as the means ± standard error (*n* = 3). LOD indicates below limit of detection. Treatments were made at anthesis (GS 60). Cytokinin abbreviations: CK (cytokinins), *t*Z (*trans*-zeatin), iP (*N*^6^-isopentenyladenine), *c*Z (*cis*-zeatin), DHZ (dihydrozeatin), R (riboside), OG (*O*-glucoside), RMP (riboside-5′-monophosphate), 7G (7-*N*-glucoside), 9G (9-*N*-glucoside).

**Table 3:** LC–MS/MS analyses of the quantity of cytokinins in barley (cultivar Quench, 2014/15) grains treated at anthesis with INCYDE. Measurements were made at four days after anthesis.

Barley Cytokinin Concentrations (pmol/g DW)		
Type	Control	INCYDE 50 µM
*t*Z	759.5 ± 66.8	642.8 ± 30.0
*t*ZR	432.0 ± 44.3	458.9 ± 36.6
*t*ZOG	89.5 ± 2.7	97.6 ± 7.9
*t*ZROG	28.1 ± 1.8	30.2 ± 0.6
*t*ZRMP	455.3 ± 27.0	454.6 ± 13.0
*t*Z7G	<LOD	<LOD
*t*Z9G	46.2 ± 2.6	65.0 ± 9.1
Total *t*Z types	1810.6 ± 142.1	1749.1 ± 61.6
iP	2.0 ± 0.2	2.3 ± 0.3
iPR	4.5 ± 0.6	4.7 ± 0.3
iPRMP	86.8 ± 12.8	89.0 ± 11.9
iP7G	<LOD	<LOD
iP9G	<LOD	<LOD
Total iP types	93.3 ± 13.6	96.0 ± 12.2
*c*Z	3.6 ± 0.1	3.2 ± 0.1
*c*ZR	**23.4 ± 0.5**	20.1 ± 0.5 *
*c*ZOG	**328.9 ± 11.8**	417.1 ± 9.7 *
*c*ZROG	242.5 ± 8.4	256.8 ± 8.6
*c*ZRMP	20.0 ± 1.8	20.6 ± 1.9
*c*Z9G	<LOD	<LOD
Total *c*Z types	618.4 ± 18.8	717.9 ± 12.8 *
DHZ	**0.9 ± 0.02**	0.7 ± 0.04 *
DHZR	**6.5 ± 0.1**	5.1 ± 0.3 *
DHZOG	**12.6 ± 0.6**	10.6 ± 0.4 *
DHZROG	47.3 ± 3.1	43.0 ± 2.8
DHZRMP	<LOD	<LOD
DHZ7G	6.0 ± 0.3	5.0 ± 0.7
DHZ9G	0.06 ± 0.01	0.06 ± 0.01
Total DHZ types	73.0 ± 3.7	64.5 ± 3.8
Total CK bases	766.0 ± 67.0	649.0 ± 30.4
Total CK ribosides	466.3 ± 44.6	488.8 ± 37.1
Total CK nucleotides	562.2 ± 38.6	564.2 ± 11.1
Total CK *O*-glucosides	**748.9 ± 27.8**	855.4 ± 25.2 *
Total CK *N*-glucosides	51.9 ± 2.6	70.1 ± 8.7
Total cytokinins	2595.2 ± 177.1	2627.5 ± 68.9

* Indicates a statistically significant (*p* ≤ 0.05) difference for the treatment compared to the control using a two-sided ANOVA and *post hoc* two-sided Dunnett test (CI: 95%). Significant differences are provided in bold. Data are presented as the means ± standard error (*n* = 3). LOD indicates below limit of detection. Treatments were made at anthesis (GS 60). Cytokinin abbreviations: CK (cytokinins), *t*Z (*trans*-zeatin), iP (*N*^6^-isopentenyladenine), *c*Z (*cis*-zeatin), DHZ (dihydrozeatin), R (riboside), OG (*O*-glucoside), RMP (riboside-5′-monophosphate), 7G (7-*N*-glucoside), 9G (9-*N*-glucoside).

## Data Availability

All data are contained within the article.
